# Navigating immigration policy and promoting health equity: Practical strategies for clinicians

**DOI:** 10.1002/jhm.12792

**Published:** 2022-02-14

**Authors:** Lilli Mann‐Jackson, Senthuran Ravindran, Alexander Perez, Julie M. Linton

**Affiliations:** ^1^ Department of Social Sciences and Health Policy, CTSI Program in Community-Engaged Research Wake Forest School of Medicine Winston‐Salem North Carolina USA; ^2^ Departments of Medicine and Pediatrics University of South Carolina School of Medicine Greenville Greenville South Carolina USA; ^3^ Department of Pediatrics Prisma Health Upstate Greenville South Carolina USA; ^4^ Office of Student Affairs and Admissions University of South Carolina School of Medicine Greenville Greenville South Carolina USA

## A VIGNETTE

A 40‐year‐old male who prefers to speak Spanish presents to the emergency room with 12 days of fever, myalgias, generalized weakness, progressive shortness of breath, and nonproductive cough. His medical history is notable for obesity, uncontrolled type 2 diabetes, and hypertension, and he currently takes no medications. His oxygen saturation on room air is 82% with a respiratory rate of 34/min. He is placed on 5 Liters nasal cannula and subsequently escalated to heated‐high flow nasal cannula. He is diagnosed with acute hypoxemic respiratory failure due to COVID‐19 pneumonia. He has not received the COVID vaccine, citing limited transportation and his work schedule as significant barriers. Numerous sick contacts include his wife and two teenage daughters, who are all unvaccinated, and his coworkers, with whom he carpools to job sites. Phone interpretation has been the only communication option throughout the hospitalization. Pending discharge, physical therapy recommends post‐acute rehabilitation but when case management inquires about his need for rehabilitation the patient discloses that he is ineligible for insurance due to his immigration status. His last primary care visit at the free clinic was more than 1 year ago.

## INTRODUCTION

A myriad of factors may shape immigrants' health prior to, during, and after hospitalization, such as for the patient in this vignette. Immigrants in the United States are highly heterogeneous in country of origin, immigration status, economic status, education, and other demographic characteristics.^1^, ^2^ Immigrants' healthcare experiences and outcomes, particularly for those who are undocumented or in mixed immigration‐status households,^3^ are deeply impacted by complex intersections between federal, state, and local policies and other social drivers that disproportionately impact some immigrant groups. A recent meta‐narrative literature review identified key social drivers of health for immigrant families, ranging from the individual patient level to public policy level,^4^ and a policy scan of all US states and major metropolitan areas yielded 539 state and 322 municipal laws and policies relevant to immigrant health.^5^ COVID‐19 has highlighted many of these obstacles for immigrant families that predate the pandemic.

Prior to hospitalization, insurance status, limited access to primary care and preventive services (e.g., chronic disease management and COVID‐19 vaccination), language barriers, inflexible work schedules, and transportation challenges may delay presentation or worsen morbidity for immigrant patients.^6^, ^7^ Immigrants who are undocumented or in mixed‐status households may fear that seeking care could expose themselves or family members to immigration enforcement.^8^ Additionally, lack of control over one's work environment and economic pressures may increase risk and detract immigrant workers from staying home from work if sick or exposed, contributing to disproportionate rates of COVID‐19 among immigrant communities.^9^ For hospitalized patients, costs may be covered by emergency Medicaid, which reimburses hospitals for providing care to individuals otherwise eligible for Medicaid except for their immigration status for conditions that without immediate medical attention could put the patient's health in jeopardy,^10^ but immigrant patients and healthcare professionals may be unaware of eligibility. After hospitalization, immigration‐related drivers of health persist, as lack of insurance and inconsistent preventive care impede adherence to follow‐up care recommendations.^11^

**Figure 1:**
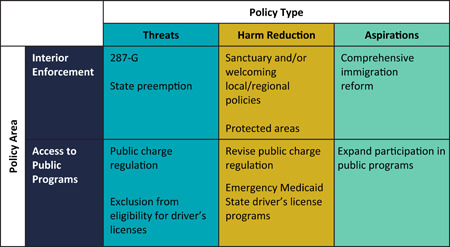
Framework for understanding immigration‐related policies that contribute to the health of immigrant patients in US hospital settings

We have identified two priority areas of immigration‐related policies that contribute to the health of hospitalized immigrant patients: interior enforcement and public programs access. Policies within these areas can be conceptualized as threats (i.e., policies that may adversely affect immigrant health), harm reduction (i.e., policies that may mitigate negative impacts of these threats), and aspirations (i.e., potential policies that, if enacted, may promote immigrant health; Figure 1). Of note, other policy areas, including the detention of newly arrived immigrants, may also impact hospitalizations,^12^, ^13^ but a detailed discussion of border policies is beyond the scope of this article. Through the lens of this vignette depicting an undocumented immigrant whose experiences may reflect those with similar lived experiences, we will explore interior enforcement and public program policy threats, harm reduction opportunities, and aspirations for advancing health equity in the context of hospital medicine.

## IMMIGRATION POLICY THREATS, HARM REDUCTION APPROACHES, AND ASPIRATIONS

### Interior enforcement

#### Threats

Policies promoting collaboration between state and local law enforcement and federal immigration enforcement threaten the health of immigrants and contribute to issues of care avoidance such as in the vignette. Such policies include Section 287(g) of the Immigration and Nationality Act, through which Immigration and Customs Enforcement enters into agreements allowing state and local law enforcement to perform immigration enforcement activities (https://www.ice.gov/identify-and-arrest/287g). Among persons from Spanish‐speaking countries in Latin America living in North Carolina, such policies created practical barriers to accessing and utilizing health services, compounded distrust of services, negatively impacted physical and mental health, and compromised child health, due to heightened fears that driving to or visiting clinics could lead to encounters with local law enforcement with potential immigration‐related consequences (e.g., initiation of deportation proceedings). These effects were felt both in counties with and without 287(g) agreements and inhibited seeking needed services across county lines, as immigrants often travel within the state to receive health care.^14^ Other researchers have documented similar effects of these policies on healthcare access and outcomes,^15^, ^16^, ^17^ including potentially avoidable pediatric hospitalizations.^18^

Similarly, state preemption involves the use of state law to restrict municipalities' authority to protect immigrants. Such laws limit the positive impacts of supportive municipal laws and policies, are confusing to both immigrants and health service providers, and have a chilling effect on the use of health services.^19^ Specifically, interior enforcement concerns may contribute to immigrants' decisions to forgo care for chronic conditions or delay seeking urgent care, which both impacted the experience of the hospitalized patient in our vignette.

#### Harm reduction

Conversely, welcoming local and regional policies, such as “sanctuary city” policies that limit local cooperation with immigration enforcement, can mitigate these threats. Though these municipal laws and policies do not eliminate challenges faced by immigrants, they can increase trust in local health services and allow immigrants to feel safer seeking those services.^20^

Harms are further reduced by federal policies preventing immigration enforcement in specific settings. Hospitals, schools, and places of worship have long been designated “sensitive locations” off‐limits for enforcement activities. The Department of Homeland Security recently expanded these “protected areas” to other places where such activities would limit access to essential services (https://www.dhs.gov/publication/guidelines-enforcement-actions-or-near-protected-areas-memo). Given the pervasiveness of fears regarding the threat of immigration enforcement, interventions to disseminate clear information about these protections are essential to promote healthcare access among immigrant patients who may need hospital services.

#### Aspirations

Ultimately, comprehensive federal immigration reform that includes a pathway to citizenship has the greatest potential to eliminate immigration enforcement‐related threats. Policies granting realistic opportunities to adjust one's immigration status would remove concerns about interior enforcement and deportation from the decision‐making process for many immigrants and their families when seeking care, and lessen the need for a patchwork of harm reduction approaches to mitigate these concerns.^21^

### Access to public programs

#### Threats

Pervasive restrictions on enrollment eligibility, such as those that prevented the patient in our vignette from qualifying for health insurance, specifically exclude undocumented people from federal public programs, including Medicaid and the Supplemental Nutrition Assistance Program (SNAP).^10^ Additionally, changes made in 2019 to the federal public charge rule (https://www.uscis.gov/archive/public-charge-fact-sheet) exacerbated existing broader restrictions on immigrants' access to public programs. Before 2019, immigrants applying for lawful permanent residency (i.e., a Green Card) could be disqualified based on previous use of cash benefits, such as Temporary Assistance for Needy Families; the changes expanded public charge to also include noncash health benefits such as Medicaid and SNAP and negatively impacted people with low incomes. Though many immigrants are ineligible for Medicaid and SNAP, these changes led to reduced use among those who were eligible, including among immigrant children, essential workers, and other special populations. Fear and confusion around public charge have decreased the use of services beyond the populations and programs included in the law, with spillover to enrollment in programs such as the Special Supplemental Nutrition Program for Women, Infants, and Children (WIC).^22^, ^23^, ^24^

State laws and policies restricting individuals without social security numbers from obtaining driver's licenses pose another profound threat to immigrants, limiting mobility and the ability to identify oneself to access services.^25^ These policies intersect with other immigration enforcement policies by increasing fears regarding potential detention or deportation if stopped for driving without a license by local law enforcement collaborating with federal immigration enforcement.^14^ For example, the patient in our vignette was excluded from accessing a driver's license, and transportation barriers impeded access to services.

#### Harm reduction

In 2021, a reversal of the 2019 public charge rule again allowed for eligible immigrants to participate in public programs. Given that generalized fear and misconceptions regarding these policies pervade, concerted efforts are needed to communicate updated information to immigrant communities and mitigate mistrust of services.

Emergency Medicaid often temporarily covers costs during hospitalization and is a rare exception to immigrant exclusion at the federal level. However, emergency Medicaid does not cover home health services, so supplemental harm reduction programs such as hospital‐based charity care are essential to mitigate the prolonged length of stay and avoid preventable readmissions. Furthermore, lack of coverage for care outside the hospital has been identified as contributing to increased costs to the healthcare system by impeding immigrants' access to routine and follow‐up care and thus leading to increased emergency department visits and hospitalizations.^26^, ^27^

Enforcement‐related fears and transportation barriers can be mitigated through state driver's license programs. As of 2020, 16 states issue driver's licenses to undocumented residents.^28^ Expanding immigrant eligibility for driver's licenses is a public health intervention that could substantially increase services access while improving public safety.^29^

#### Aspirations

Opportunities exist, particularly at the state level, to disrupt the conflation of immigration status with access to basic healthcare. For example, six states have already opted to provide medical coverage for children regardless of immigration status and 20 have done so for pregnant persons; California has further expanded its state Medicaid program to include undocumented young adults and older adults.^30^, ^31^ Expanded coverage has the potential to facilitate immigrants' abilities to receive needed care prior to, during, and after hospitalization.

## RECOMMENDATIONS

Amidst policies ranging from restrictive to harm reductionist to aspirational across different regions and settings, clinicians can engage in key strategies to advance delivery of high‐quality, accessible, and equitable care prior to, during, and after hospitalization.

1. *Offer training for social workers, case managers, and financial counselors* to cover policy‐related topics such as chilling effects of the public charge rule, emergency Medicaid eligibility, and medication access.
2. *Engage community and partner with community health workers familiar with the larger policy context* to connect families to key resources and deliver key health messages.
3. *Expand rounding strategies* to incorporate “social barriers to care” (including those related to immigration) into inpatient problem lists and progress note templates, add a “transitions of care” section to discharge summary templates to ensure follow‐up care, and intentionally recognize limitations in access to public health benefits, and coordinate rounding on patients who prefer languages other than English to optimize the use of in‐person interpreters.
4. *Model nuanced, culturally compassionate care delivery* for learners and incorporate immigrant health into longitudinal didactic curricula to actively engage all learners, not just those already invested in the work. Create welcoming spaces for dialogue and seek opportunities to teach through a public health lens. Discontinue the use of language that places blame on people who have experienced oppression, using instead language that acknowledges structural racism and inequitable policy.
5. *Recruit, mentor, and sponsor bilingual, bicultural learners* to build a workforce prepared to meet the needs of diverse communities.
6. *Share de‐identified stories*, with patient and family permission, to advocate for equitable policy change.
7. *Collaborate* to engage with professional organizations and across sectors (e.g., legal, education, and mental health) to advocate for inclusive local, state, and federal programs and policy aspirations.

## CONCLUSION

Immigration status is a core social driver of health. Patients' experiences elucidate how restrictive policies threaten health, galvanizing advocacy efforts to advance equitable immigration policy at local, state, and federal levels. Clinicians have opportunities to engage in clinical and advocacy interventions that acknowledge the implications of such policies on access to and participation in programs that optimize health and wellbeing.

## CONFLICT OF INTEREST

The authors declare that there are no conflicts of interest.
